# A Cys2His2 Zinc Finger Transcription Factor *BpSZA1* Positively Modulates Salt Stress in *Betula platyphylla*

**DOI:** 10.3389/fpls.2022.823547

**Published:** 2022-05-25

**Authors:** Xi Zhang, Qing Guo, Linlin Qin, Li Li

**Affiliations:** State Key Laboratory of Tree Genetics and Breeding, Northeast Forestry University, Harbin, China

**Keywords:** *Betula platyphylla*, *BpSZA1*, salt stress, ChIP, ROS scavenging

## Abstract

Zinc finger proteins (ZFPs) are widely involved in plant growth and abiotic stress responses, however, few of these proteins have been functionally characterized in tree species. In this study, we cloned and characterized the *BpSZA1* gene encoding a C2H2-type ZFP from *Betula platyphylla*. *BpSZA1* is a transcription factor localized in the nucleus, with a transcription activation domain located at the N-terminus. *BpSZA1* was predominantly expressed in stems and was induced by salt. We generated transgenic birch lines displaying overexpression (OE) or RNAi silencing (Ri) of *BpSZA1* and exposed these along with wild-type birch seedlings to salinity. Phenotypic and physiological parameters such as superoxide dismutase, peroxisome, H_2_O_2_ content, proline content, water loss rate, and malondialdehyde content were examined. Overexpression of *BpSZA1* in birch conferred increased salt tolerance. Chromatin immunoprecipitation-qPCR and RNA-seq showed that BpSZA1 binds to the GAGA-motif in the promoter of downstream target genes including *BpAPX1*, *BpAPX2*, *BpCAT*, and *Bp6PGDH* to activate their transcription. *BpSZA1* also participates in abscisic acid (ABA) biosynthesis, proline biosynthesis, and the ABA/jasmonic acid pathway to enhance the salt stress of *B. platyphylla*.

## Introduction

Plants typically suffer multiple abiotic stresses during their life cycle, which limit their growth and development. Climate extremes and modern agricultural practices are leading to continuous increases in soil salinity, making this a major abiotic stress (Abe et al., 2003; Franco-Zorrilla et al., 2014; Shrivastava and Kumar, 2015; Zhu, 2016). High salinity causes osmotic salt stress by reducing water uptake; it also accelerates the production of active oxygen radicals, leading to oxidative bursts that can damage or even kill plants (Munns and Tester, 2008; Franco-Zorrilla et al., 2014). To cope with salt stress, plants have evolved various defense mechanisms. Organic solutes including sucrose, proline, and glycinebetaine, accumulate in the cytosol and organelles to balance the osmotic potential of vacuolar Na^+^ (Hasegawa et al., 2000; Seki et al., 2003). In addition, antioxidant enzymes such as superoxide dismutase (SOD), peroxidase (POD), ascorbate peroxidase (APX), and catalase (CAT) and non-enzymatic compounds (ascorbate and reduced glutathione) are essential for reactive oxygen species (ROS) homeostasis, detoxifying ROS and increasing ROS scavenging ability (Franco-Zorrilla et al., 2014; Gupta and Huang, 2014). Those defense mechanisms are known to be regulated by specific transcription factors (TFs) including APETALA2/ethylene-responsive factor (AP2/ERF), v-myb avian myeloblastosis viral oncogene homolog (MYB), WRKY, basic region-leucine zipper (bZIP), and zinc finger proteins (ZFPs) (Riechmann et al., 2000; Seki et al., 2003; Guo et al., 2005; Iida et al., 2005; Wang et al., 2019). Thus, TFs serve as important participants in plant responses to salt stress by combining with various *cis*-elements in the promoter regions of downstream genes to modify their expression (Abe et al., 2003; Franco-Zorrilla et al., 2014).

Zinc finger proteins are found widely in animals, plants, and microorganisms (Miller et al., 1985). Plant ZFPs are divided into nine subfamilies, C2H2, C2HC, C4, C6, C8, C2HC5, C3HC4, C4HC3, and CCCH depending on the number and position of cysteine (Cys) and histidine (His) residues (Berg and Shi, 1996). Plant C2H2 type ZFPs from one of the largest subfamilies of ZFPs and play a crucial role in plant development (Englbrecht et al., 2004; Takatsuji, 1998). STAMENLESS 1, a C2H2 ZFP from rice, positively modulated floral organ identity by participating in transcriptional regulation of SPW1/OsMADS16 (Xiao et al., 2009). Also in rice, NON-STOP GLUMES1 regulated Spikelet development by inhibiting the expression of DROOPING LEAF (DL), LONGSTERILELEMMAS 1 (G1), and MOSAIC FLORAL ORGANS 1 (MFO1) (Zhuang et al., 2020). In wheat, Tipped1, containing an EAR motif, acts as a transcriptional repressor to regulate awn development; lines with a high expression level of *Tipped1* displayed awn inhibition (Huang et al., 2020). Several studies also suggest that plant C2H2 ZFPs are involved in responses to salt stresses (Kiełbowicz-Matuk, 2012; Wang et al., 2019). For example, the heterologous expression of *ZjZFN1* enhances the salt tolerance of transgenic *Arabidopsis* plants (Teng et al., 2018). Tomato SlZF3 interacts with CSN5B to confer greater tolerance to salt treatment in lines overexpressing *SlZF3* (Li et al., 2018). OsZFP213, a C2H2 ZFP from wheat, confers salt tolerance by interacting with OsMAPK3 (Zhang et al., 2018). In a recent study, overexpression of *GhZAT34* and *GhZAT79* in cotton enhanced salt tolerance (Rehman et al., 2021). Taken together, these studies demonstrate that C2H2 ZFPs play an important role in plant development and the response of plants to salt stress.

To screen differentially expressed genes (DEGs) at the transcription level, RNA-seq can be used and requires neither genome annotation nor pre-synthesized nucleotides as probes. More and more studies have identified genes differentially expressed under abiotic stress using RNA-seq. Evidence suggests that there are 97 putative WRKY proteins in pearl millet, with *PgWRKY33*, *PgWRKY62*, and *PgWRKY65* responding to dehydration and salinity stress (Chanwala et al., 2020). Similarly, 132 NAC proteins were identified in peanuts using RNA-seq; these were classified into eight subgroups, where the members of groups IV, VII, and VIII are induced by drought and abscisic acid (ABA) stresses (Li et al., 2021). In addition, genes encoding 197 bHLH proteins have been identified in pear, and *PbrbHLH7*, *PbrbHLH8*, *PbrbHLH128*, *PbrbHLH160*, *PbrbHLH161*, and *PbrbHLH195* are up-regulated under cold and drought stress (Dong et al., 2021). RNA-seq is thus a useful tool for identifying TFs that respond to abiotic stress.

Chromatin immunoprecipitation (ChIP) is used to detect interactions between TFs and DNA *in vivo* and can also identify downstream genes regulated by TFs (Nelson et al., 2006). Recent work established that, in abiotic stress, WRKY33 and WRKY12 can form a complex under abiotic stress increasing the expression of *RAP2.2* to enhance hypoxia tolerance in *Arabidopsis* (Tang et al., 2021). Similarly, the ChIP-seq assay combined with RNA-seq revealed that *SlGRAS4* forms a homodimer to activate its transcription as well as bind directly to the *SlCBF* promoter to enhance cold tolerance (Liu et al., 2020). *ZmNST3*, a member of the NAC TF family, directly combines with the promoter of *GST/GlnRS* to activate its transcription and increase ROS scavenging, thereby enhancing drought tolerance in maize (Ren et al., 2020). Currently, however, there are limited reports on the application of ChIP technology to forests species.

Birch (*Betula platyphylla* Suk.) is widely distributed in cool-temperate and boreal forests in the northern hemisphere (Kazuhiko, 2007) and is widely used in building and wooden furniture (Li et al., 2002). However, birch is more sensitive to salt than *Ulmus pumila* and *Fraxinus mandshurica*. So it is important to study the salt-tolerance mechanisms of *B. platyphylla* for cultivating salt-tolerant birch varieties. In this study, we cloned a gene encoding a C1-2i subfamily member of the C2H2 ZFPs from *B. platyphylla* that is induced by salt. The gene was named *BpSZA1*, and we generated lines overexpressing (OE) or showing RNAi silencing (Ri) lines of *BpSZA1* for gain- and loss- of function analysis. *BpSZA1* conferred increased tolerance to salt stress in OE lines compared with wild-type (WT) and Ri lines by participating in ROS scavenging, ABA biosynthesis, proline biosynthesis, and jasmonic acid (JA) responses. Our results provide useful information on the function of C2H2 ZFPs in response to abiotic stress.

## Materials and Methods

### Plant Materials and Growth Conditions

Birch (*B. platyphylla* Suk.) plantlets were cultured *in vitro* on solid Lloyd and McCown’s Woody Plant Basal Salts (WPM) medium as previously described (Wang et al., 2016). After regeneration, plantlets were transplanted into individual pots containing a mixture of perlite/soil/vermiculite (1:4:1, by vol) for cultivation for 8 weeks in a greenhouse under controlled conditions (16/8 h light/dark, 25 ± 1°C, and 70–75% relative humidity) for tissue-specific expression and salt stress. For tissue-specific expression, the following tissues were harvested: including YL: young leaves, leaves from the first to second internodes; AL: adult leaves, leaves from the third to fourth internodes, OL: old leaves, leaves from the fifth to sixth internodes; AS: adult stems, stems with the third to fourth internodes; OS: old stems, stems with the fifth to sixth internodes. For salt stress, the root of 8-week-old birch plants prepared as described above was watered directly with 200 mM NaCl solution, and samples were harvested after treatment for 0, 6, 12, 24, 48, and 72 h. Three independent biological replicates were performed. Leaves from the birch seedlings were harvested for RNA isolation.

### RNA Isolation and cDNA Synthesis, qRT-PCR

Total RNA was isolated from each sample using a Universal Plant Total RNA Extraction Kit according to the manufacturer’s instructions (BioTeke Corporation, China). qRT-PCR analysis, assays were performed using an SYBR Premix Ex Taq II (TaKaRa, Dalian, China) kit according to the manufacturer’s specifications. Quantification was performed using the 2^–ΔΔt^ method, where the birch Tubulin gene *BpTubulin* is a housekeeping gene for normalization (Regier and Frey, 2010). All primers used in this study are detailed in Supplementary Table 1.

### Bioinformatics Analysis of *BpSZA1*

A previously released dataset of *B. platyphylla* (Wang et al., 2018) was analyzed to identify C2H2 ZFP genes exhibiting induced by salt stress. Birch_GENE_10017081 was selected for further analysis because its expression was upregulated at all-time points. Protein information was obtained from the amino acid sequence encoded by the gene and was analyzed using ExPASY.^1^ The ZFP sequences in *Arabidopsis*, *Populus pilosa*, and *Betula pendula* subsp. were obtained from TAIR,^2^ the phytozome database,^3^ and the CoGe database,^4^ respectively. A phylogenetic tree was reconstructed in MEGA7 using the maximum likelihood method with 1,000 bootstraps replicates. Conserved motifs in *BpSZA1* were predicted using Multiple Expectation maximizations for Motif Elicitation (MEME^5^).

### Binary Vector Generation and Plant Transformation

Overexpression and RNAi constructs were generated using the BP and LR Gateway cloning system following the manufacturer’s instructions (Thermo Fisher Scientific, United States). The full-length of *BpSZA1* CDS (open reading frame) without termination codon was amplified using gene-specific primers for the overexpression construct. To generate the RNAi (silencing) vector, a pair of primers flanking the CDS sequence from position 532–741 bp was used. Amplified fragments were first cloned into the pENTR™/SD/D-TOPO™ vector using BP-cloning and then into the binary vectors pGWB5 (overexpression) or pB7GWIWG2 (I) through LR reactions (Karimi et al., 2002). After sequence verification, the binary plasmids were transformed into *Agrobacterium tumefaciens* strain EHA105 using the freeze/thaw method. *Agrobacterium*-mediated transformation was performed as previously described (Holsters et al., 1978). Transgene presence was verified using the PCR primers LR-35S-F(CCTCGGATTCCATTGCCCAGCTA) and LR-GFP R(GTCGATGCCCTTCAGCTCGAT). Sequences of all primers used for the cloning are shown in Supplementary Table 2.

### Subcellular Localization Assay

The full-length *BpSZA1* coding sequence (without the stop codon) was amplified and cloned into a pBI121-eGFP vector upstream of the eGFP sequence and behind the CaMV 35S promoter, according to the reading frame, to generate 35S:BpSZA1-GFP. The method of tobacco subcellular localization is based on the method published by Sparkes et al. (2006). The 35S:BpSZA1-GFP plasmid was transferred to *A. tumefaciens* GV3101 competent cells and transformed into *Nicotiana benthamiana*; 35S-GFP was used as a control. After 2–3 days, fluorescence in the tobacco cells was observed at 488 nm under a confocal laser scanning microscope LSM700 (Zeiss, Jena, Germany). Sequences of all primers used for the cloning are shown in Supplementary Table 3.

### Transactivation Assay

To examine the transactivation activity of *BpSZA1* and determine the minimal domain required for activation, the complete *BpSZA1* CDS along with seven truncated *BpSZA1* fragments were individually fused to the GAL4 DNA binding domain in the pGBKT7 vector using the In-Fusion HD Cloning System CE (TaKaRa, Dalian, China). The recombinant plasmids and the pGBKT7 vector (negative control) were individually transformed into yeast strain Y2HGold. Transformants were successfully dropped onto SD/-Trp and SD/-Trp/-His/-Ade/X-α-gal selective medium and incubated at 30°C for 3–5 days. The transactivation activity was evaluated according to the growth status of colonies and the blue color manifested by X-α-gal activity. Sequences of all primers used for cloning are shown in Supplementary Table 4.

### Salt Stress Treatments

For salt stress treatments, 8-week-old WT and transgenic *B. platyphylla* seedlings, three OE lines (OE2, OE6, and OE9) and three Ri lines (Ri5, Ri7, and Ri8), were treated with 200 mM NaCl for 7 days and the phenotypes were photographed.

For plant height, fresh weight, and root length measurements, 7-day-old birch seedlings (WT, OE2, OE6, OE9, Ri5, Ri7, and Ri8) were cultured in half MS minimal medium containing 40 mM NaCl. After 4 weeks of growth, plant height, fresh weight, and root length were measured. Birch seedlings grown in half MS minimal medium were used as a control.

### Analysis of Physiological Parameters

Similar-sized birch seedlings were treated with 200 mM NaCl for 3 days, and adult leaves (WT, OE6, OE9, Ri5, and Ri7) were harvested. SOD activity was measured using a SOD test kit (Nanjing Jiancheng Bioengineering Institute, A001-1), POD activity was measured using a POD test kit (Nanjing Jiancheng Bioengineering Institute, A084-3), malondialdehyde (MDA) content was measured as described previously (Heath and Packer, 1968), proline content was determined according to a previously published protocol (Bates, 1973), and total chlorophyll content was assayed using a method previously published (Minotti et al., 1994). For electrolyte leakage analysis, leaf samples of *B. platyphylla* were collected using a 1-cm punch with 10 pieces for each sample, as previously described (Ben-Amor et al., 1999). Leaves of *B. platyphylla* were treated with 200 mM NaCl for 6 h, and leaves were then detached and stained with 3,3-diaminobenzidine (DAB) or nitro blue tetrazolium (NBT) solution to detect H_2_O_2_ or O_2_^2–^, respectively, according to a previously published method (Fryer et al., 2002).

### Transcriptome Determination and Analysis

Leaves of 8-week-old *B. platyphylla* OE5, Ri7, and WT seedlings treated with 200 mM NaCl were rapidly collected after 3 days of treatment. Six samples with three repeats were used for RNA sequence analysis—Ri before/OE before, Ri before/WT before, WT before/OE before, Ri after/OE after, Ri after/WT after, and WT after/OE after—where “before” indicates samples collected before salt stress and “after” indicates samples collected after salt stress. A total of 1 μg RNA per sample was used as input material for RNA sample preparations. Sequencing libraries were generated using a NEBNext Ultra™ RNA Library Prep Kit for Illumina (NEB, United States) following the manufacturer’s recommendations. Clustering of index-coded samples was performed on a cBot Cluster Generation System using a TruSeq PE Cluster Kit v4-cBot-HS (Illumina) according to the manufacturer’s instructions. After cluster generation, library preparations were sequenced on an Illumina platform and paired-end reads were generated. Adaptor sequences and low-quality sequence reads were removed from the data sets. Raw sequences were transformed into clean reads after data processing. Hisat2^6^ tools software was used to map reads to the reference birch genome.^7^ Transcripts were assembled using StringTie.^8^ Differential expression analysis of pairs of samples was performed using EBseq.^9^ The false discover rate (FDR) < 0.01 and |log2(fold change)| ≥ 2 were set as the threshold for significantly differential expression. Gene Ontology (GO) enrichment analysis of DEGs was implemented using the GOseq R packages based on Wallenius non-central hypergeometric distribution (Young et al., 2010), which can adjust for gene length bias in DEGs.

### Chromatin Immunoprecipitation-PCR and Chromatin Immunoprecipitation-qPCR Analysis

To identify putative target genes of *BpSZA1*, ChIP-PCR, and ChIP-qPCR were used. Sonication conditions of the samples were optimized according to a previously described method (Li et al., 2014). Briefly, approximately 2 g pGWB5-BpSZA1-OE birch seedlings were immersed in 1% (w/v) formaldehyde for protein cross-linking and protein binding to DNA. Next, ChIP was followed by extraction and shearing of the chromatin precipitated using a ChIP analysis kit (P2078, Beyotime) according to the manufacturer’s instructions. An anti-GFP antibody (AG281, Beyotime) (ChIP+) was then used and chromatin was immunoprecipitated; an anti-IgG2a antibody was used as a negative control (Mock). Chromatin without immunoprecipitation was used as the input control (Input). Three biological replicates were carried out. All primers used for ChIP-PCR and ChIP-qPCR analysis are detailed in Supplementary Table 6.

### Statistical Analysis

All experiments were conducted with at least three biological replicates unless otherwise mentioned, and the standard error of the mean was computed in each case. The Student’s *t*-test was performed for the estimation of statistical significances (SPSS 18; IBM Corp., Armonk, NY, United States). Data points representing statistically significant differences between WT and transgenic lines or between control and stress conditions are indicated.

## Results

### Identification and Structural Analysis of *BpSZA1*

**FIGURE 1:**
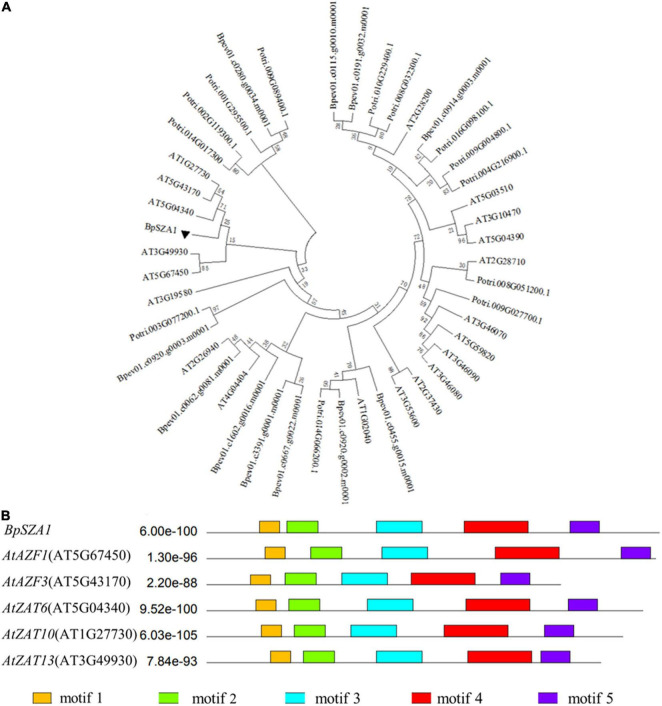
Phylogenetic and bioinformatics analyses of *BpSZA1* from *Betula platyphylla*. **(A)** Phylogenetic analysis of BpSZA1 and 45 members of the C2H2-type transcription factor from *Arabidopsis thaliana* (20 members, prefixed with At), *Populus trichocarpa* (13 members, prefixed with Potri), and *Betula pendula Roth* (12 members, prefixed with Bpev) by using the maximum likelihood method. **(B)** Conserved protein motifs of *BpSZA1* and five C2H2-type transcription factor C1-2i subfamily members in *Arabidopsis thaliana*.

**FIGURE 2:**
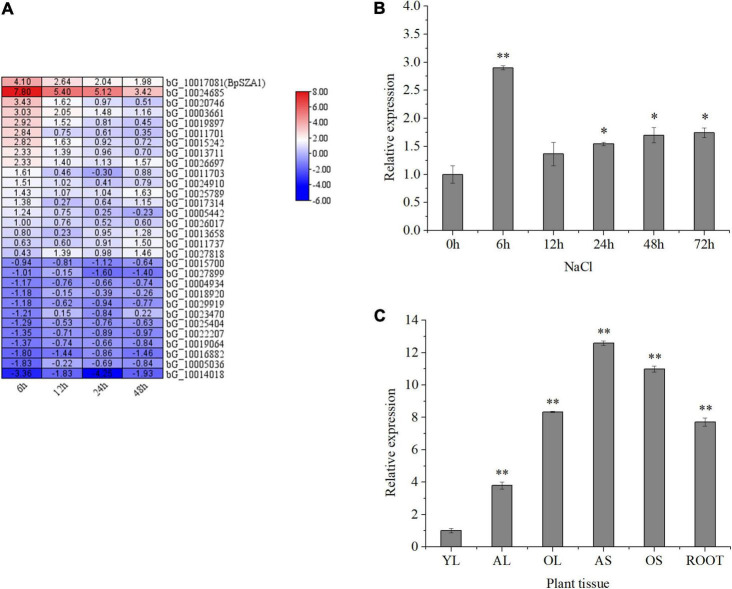
Expression of genes encoding zinc finger proteins in *B. platyphylla* under 200 mM NaCl salt stress, and *BpSZA1* expression patterns in different tissues and under different treatments. **(A)** Bioinformatic analysis of the transcriptome data set showing expression levels for 30 genes encoding zinc finger proteins that were upregulated and downregulated after exposure of birch to 200 mM NaCl for 6, 12, 24, and 48 h. The color scale represents change. **(B)** Transcript levels of *BpSZA1* in different tissues of *B. platyphylla*. YL, young leaf; AL, adult leaf; OL, old leaf; AS, adult stem; OS, old stem; R, root. **(C)** Relative expression levels of *BpSZA1* were measured by RT-qPCR in response to NaCl. Values are mean ± SE (*n* = 15). Asterisks denote significant differences: *P ≤ 0.05; ^**^P ≤ 0.01. Error bars represent standard error for three replicates.

To identify valuable salt-induced genes from *B. platyphylla* and to cultivate salt-tolerant birch varieties, we used a next-generation sequencing technique were used to construct a cDNA library of *B. platyphylla* (200 mmol L^–1^ NaCl) (Wang et al., 2018). Analysis of RNA-seq data revealed 30 DEGs annotated as C2H2 ZFPs, of which 12 were down-regulated and 20 were up-regulated by salt, respectively. Among these salt-induced C2H2 ZFPs, birch_GENE_10017081, named *BpSZA1* (GenBank ID: MZ544846), was steadily up-regulated at all-time points under salt stress compared with the control condition (Figure 2A). We used PCR to amplify the cDNA of *BpSZA1*, and sequencing results indicated that the full-length *BpSZA1* is 744 bp without introns, encoding a 247 aa protein with a predicted mass of 26.24 kDa and a theoretical pI of 8.66. A phylogenetic tree was reconstructed using nucleotide sequences of 20 members of the ZFPs family from *Arabidopsis thaliana* and 13 from *Populus trichocarpa* available in the Phytozome database and 12 genes from *B. pendula* subsp. available in the CoGe database. *BpSZA1* was found to cluster with *AtAZF2* (AT3G19580), *AtAZF3* (AT5G43170), *AtZAT6* (AT5G04340), *AtZAT10* (AT1G27730), and *AtZAT13* (AT3G49930) from *Arabidopsis* (Figure 1A). Furthermore, we used these six sequences to predict conserved motifs using MEME, identifying the presence of five conserved motifs (Figure 1B). There were two zinc finger domains of QALGGH among the five motifs (motif 1 and motif 3). Specific motif sequences are shown in Supplementary Table 7.

### Expression Analysis of BpSZA1

To verify the accuracy of RNA-seq, we treated birch seedlings with NaCl and examined *BpSZA1* transcript levels. Under salt treatment (NaCl) (Figure 2C), *BpSZA1* expression increased over the first 6 h, showing a 3.48-fold increase compared with expression at 0 h, and then gradually declined. This result verified the accuracy of RNA-seq and indicated that the expression of *BpSZA1* is responsive to salt stress.

To investigate the spatial expression of *BpSZA1*, we used RT-PCR in different tissues (Figure 2B). *BpSZA1* was ubiquitously expressed in all tissues, with the highest expression levels in adult stems, followed by old stems, roots, old leaves, and adult leaves. Expression levels of *BpSZA1* were lowest among all sampled tissues in young leaves, which were used as a control. These results indicated that *BpSZA1* is expressed specifically in different tissues.

### Subcellular Localization of BpSZA1 Protein

**FIGURE 3:**
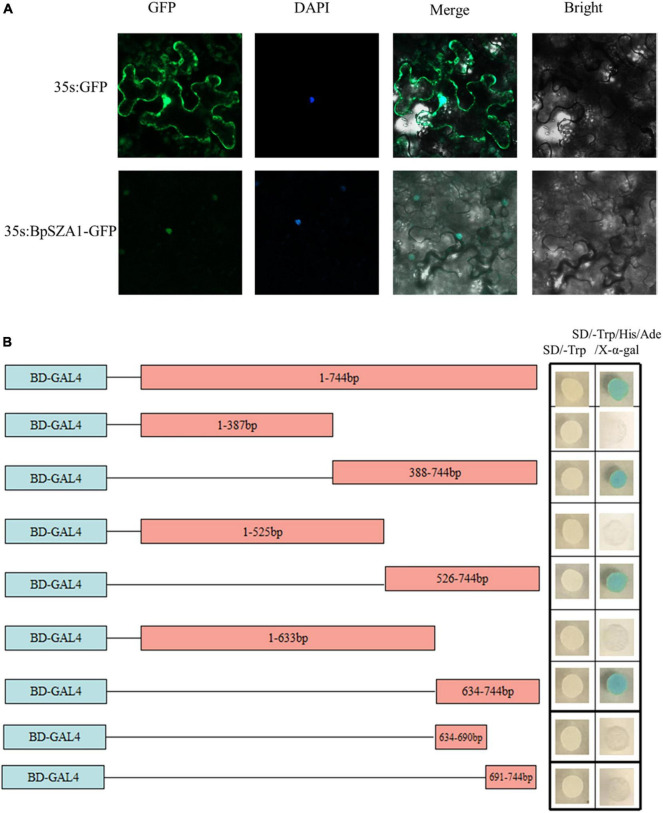
Subcellular localization and transactivation of BpSZA1. **(A)** Subcellular localization of BpSZA1 in transiently expressed tobacco leaves shows BpSZA1 is targeted to the nucleus. Bars = 50 μm. **(B)** Truncated sequences of the CDS of BpSZA1 were fused in-frame, respectively, to the GAL4 DNA-binding domain in pGBKT7 and transformed into Y2H yeast cells. The transformed cells were plated onto SD/-Trp (growth control) or SD/-Trp/-His/-Ade/X-a-Gal medium. pGBKT7 empty vector was used as a control.

To determine whether the C2H2 TF BpSZA1, is localized to the nucleus, we constructed the fusion vector 35s:BpSZA1: GFP and transfected this into tobacco epidermal cells, with the 35: GFP vector used as a control. Fluorescence signals in tobacco epidermal cells were observed under an LSM700 confocal laser microscope. The 35s:BpSZA1: GFP fusion protein was mainly visible in the nucleus, while the control (35s: GFP) was observed throughout the cells (Figure 3A). Hence, BpSZA1 is localized to the nucleus.

### Transcriptional Activity of BpSZA1

Transactivation activity of BpSZA1 was examined using a series of deletions of the *BpSZA1* CDS fused with the GAL4 DNA-binding domain sequence in the pGBKT7 vector and transformed into Y2H Gold cells. Cells containing the full-length CDS of *BpSZA1* grew equally well in both SD/-Trp (control) and SD/-Trp/-Leu/-Ade/X-a-Gal medium but transformed yeast cells containing the full-length *BpSZA1* CDS turned blue in SD/-Trp/-Leu/-Ade/X-a-Gal medium, indicating the BpSZA1 is a TF. Meanwhile, assays using the deletion mutants showed that the transcription activation domain of the BpSZA1 protein is located between amino acids 212aa and 247aa at the C-terminus of the protein (Figure 3B).

### Overexpression of BpSZA1 Enhanced Salt Tolerance

**FIGURE 4:**
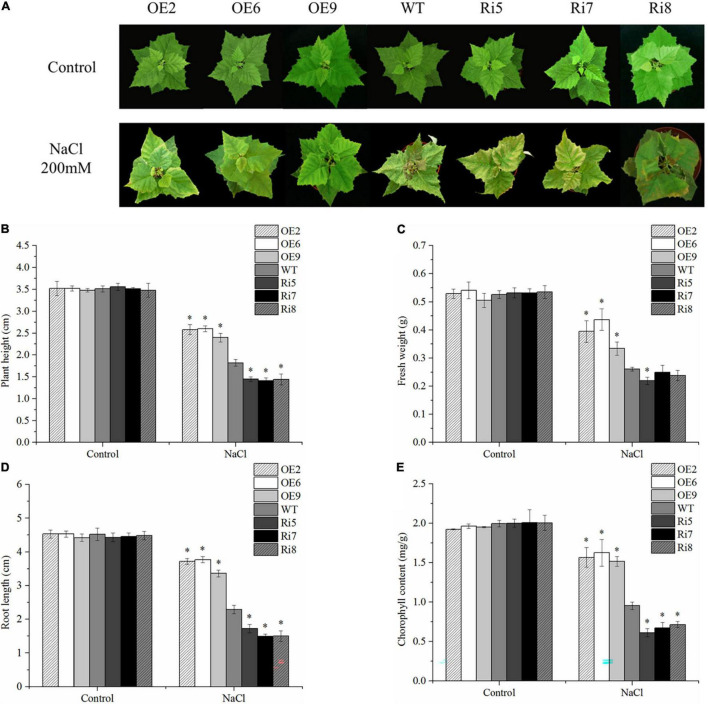
*BpSZA1* confers salt stress tolerance. **(A)** Phenotypic differences following salt treatment. **(B)** Plant height. **(C)** Fresh weight. **(D)** Relative root length. **(E)** Chlorophyll content. Asterisks denote significant differences: *P ≤ 0.05. Error bars represent standard error for three replicates.

To compare their respective stress tolerance, 8-week-old WT and OE2, OE6, OE9, Ri5, Ri7, and Ri8 transgenic birch plants grown in soil, were exposed to 200 mM NaCl. Under control conditions, there were no significant differences in the appearance of OE lines, Ri lines, and WT plants (Figure 4A). However, after 7 days of salt stress, leaves of the Ri lines showed obvious withering and yellowing compared with their appearance before stress. Obvious leaf curling and wilting also appeared in the WT seedlings, but the leaves were greener and more stretched than those of the Ri lines. By contrast, the OE lines showed only a slight curl of the leaves under salt stress, and the leaves became pale (Figure 4A). Meanwhile, there were no significant differences in plant height, fresh weight, or root length in the different lines under control conditions (Figures 4B–D), indicating that *BpSZA1* does not affect plant growth or development. Under salt stress, the plant height, fresh weight, and root length of Ri lines decreased by an average of 59, 56, and 63%, respectively, compared with measurement before stress with 48, 50, and 49% average decrease, respectively, in the WT. The reduction in plant height, fresh weight, and root length of OE lines were minimal under salt stress, being 28, 26, and 29%, respectively (Figures 4B–D). To compare growth under salt stress, we analyzed chlorophyll contents among all salt-treated lines. Under control conditions, all salt-treated lines had approximately equal chlorophyll contents (Figure 4E). However, after salt treatment, OE lines had higher chlorophyll contents compared with WT plants, but Ri lines had lower chlorophyll contents (Figure 4E). Therefore, overexpression of *BpSZA1* improved the tolerance of birch to salt stress, while inhibition of *BpSZA1* expression reduced the tolerance of birch to salt stress.

### *BpSZA1* Decreases Membrane Lipid Peroxidation and Increases Proline Content Under Salt Stress Conditions

**FIGURE 5:**
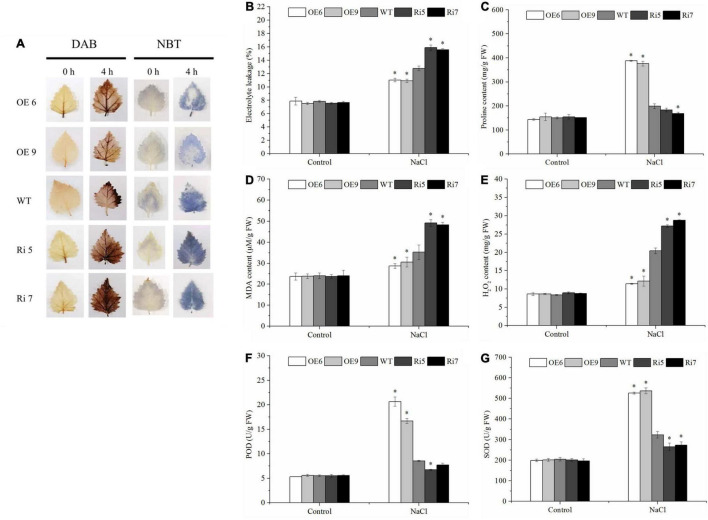
Determination of malondialdehyde (MDA) content, electrolyte leakage, proline content, MDA content, ROS accumulation (Histochemical staining and H_2_O_2_ content), superoxide dismutase (SOD) activity, peroxidase (POD) activity. Birch plantlets were treated with 200 mM NaCl for 0 h (control) and 6 h, and then their leaves were detached and stained with NBT **(A)** and DAB **(B)** to detect H_2_O_2_ and O_2_^–^. **(B)** Electrolyte leakage, **(C)** proline content, **(D)** MDA content, **(E)** H_2_O_2_ content, **(F)** SOD activity, and **(G)** POD activity in the leaves of WT, BpSZA1 OE, and Ri plants under normal, salt stress. Asterisks denote significant differences: *P ≤ 0.05. Error bars represent standard error for three replicates.

To further determine differences in salt tolerance among the WT and OE2, OE6, OE9, Ri5, Ri7, and Ri9 transgenic birch plants, we measured electrolyte leakage, MDA content, and proline content. Under control conditions, there was no significant difference in electrolyte leakage rates among WT and transgenic lines. Nonetheless, after salt stress, however, electrolyte leakage rates were increased in all salt-treated lines. Electrolyte leakage rates of WT plants were significantly lower than those of Ri lines but were significantly higher than those of OE lines (Figure 5B). Meanwhile, there was no difference in MDA content among birch lines under control conditions. When exposed to salt stress conditions, MDA contents were increased in all lines; however, OE lines had the lowest MDA contents, followed by WT, with Ri lines having the highest MDA contents (Figure 5D). Similarly, the proline content of OE lines was greater than that of WT plants under salt stress conditions; however, the proline content of Ri lines was less than that of WT plants (Figure 5C). These results suggested that compared with WT plants, OE lines showed increased salt tolerance, and Ri lines demonstrated decreased salt tolerance.

### *BpSZA1* Increases Reactive Oxygen Species Scavenging and Proline Content Under Salt Stress

We used DAB and NBT *in situ* staining to measure the accumulation of H_2_O_2_ and O_2_^2–^. Staining results revealed that under control conditions, OE, WT, and Ri lines had similar H_2_O_2_ and O_2_^2–^ levels (Figure 5A). However, when exposed to salt stress conditions, we visualized stronger staining by NBT (for O_2_^–^) or DAB (for H_2_O_2_) in Ri lines compared with WT, whereas OE lines showed less accumulation of H_2_O_2_ and O_2_^2–^ than WT plants (Figure 5A). To further clarify whether the histochemical staining differences were caused by ROS scavenging ability, we compared H_2_O_2_ content and the activities of some ROS scavenging enzymes, including POD and SOD, among the WT, OE, and Ri lines. After salt treatment, Ri lines showed higher H_2_O_2_ accumulation compared with WT plants, while OE lines had lower H_2_O_2_ accumulation (Figure 5E). However, there was no remarkable difference in H_2_O_2_ accumulation among OE lines, Ri lines, or the WT under control conditions (Figure 5E). At the same time, under control conditions, there was no difference in SOD or POD activities among any of the lines. However, when OE, Ri, and WT plants were treated with 200 mM NaCl, both SOD and POD activities were significantly lower in Ri lines compared with the WT but were significantly higher in the OE lines (Figures 5F,G). Taken together, these results indicated that overexpression of *BpSZA1* increases ROS scavenging capability under salt stress.

### Determination of the Genes Regulated by *BpSZA1* on the Genome Level

To identify genes regulated by *BpSZA1*, we performed global transcriptomic profiling by RNA-seq on OE6, WT, and Ri7 lines. Plants were untreated plants (0 day) or salt-treated for 3 days (ST 3 days). The complete list of DEGs identified by pairwise comparison is given in Supplementary Table 8. Under normal growth conditions, 884 genes were up-regulated and 716 genes were down-regulated by *BpSZA1* expression (with a *P*-value < 0.01 adjusted by the FDR) (Supplementary Figure 1A), whereas 1,119 genes were up-regulated and 1,841 genes were down-regulated by *BpSZA1* expression under salt stress condition (with a *P*-value < 0.01 adjusted by the FDR) (Supplementary Figure 1B).

Gene Ontology term analysis revealed that, before salt stress, oxidation-reduction process, protein phosphorylation, and metabolic process were enriched among biological process GO terms. Among cellular components GO terms, integral components of membrane, nucleus, and plasma membrane terms were enriched. Among molecular function GO terms, ATP binding, metal ion binding, and protein serine/threonine kinase activity were enriched. After salt stress, oxidation-reduction process, protein phosphorylation, and metabolic process were also mainly enriched among biological process GO terms. Meanwhile, response to oxidative stress and salt stress terms were also enriched among biological process GO terms. Among cellular components GO terms, integral components of membrane, plasma membrane, and membrane terms were enriched. For molecular function GO terms, ATP binding and heme binding, and metal ion binding terms were enriched, meanwhile, oxidoreductase activity and peroxidase activity were also showed genes enrichment (Supplementary Figure 2). These results indicated that genes associated with these GO terms might be regulated by *BpSZA1* in response to salt stress.

**FIGURE 6:**
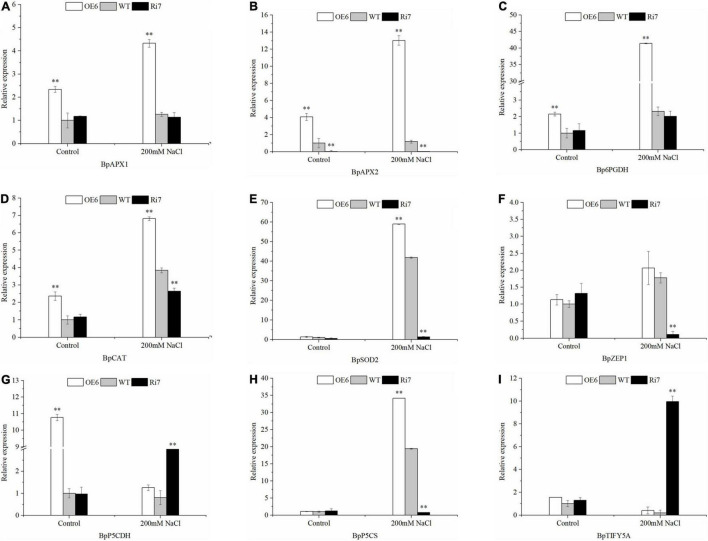
RT-PCR analysis of the expression levels of salt-responsive genes in Ri lines, WT, and OE lines before and after salt stress. **(A)** *BpAPX1*, **(B)** *BpAPX2*, **(C)** *Bp6PGDH*, **(D)** *BpCAT*, **(E)** *BpSOD2*, **(F)** *BpZEP1*, **(G)** *BpP5CDH*, **(H)** *BpP5CS*, and **(I)** *BpTIFY5A*. Asterisks denote significant differences: ^**^P ≤ 0.01. Error bars represent standard error for three replicates.

To identify the genes modulated by BpSZA1, we analyzed the expression levels of putative downstream genes in OE5, Ri7, and WT using RT-PCR. We selected the following genes according to physiological parameters and analysis of transcriptome data: *BpSOD2*, *BpAPX1*, *BpAPX2*, pyrroline-5-carboxylate synthase (*BpP5CS*), delta-1-pyrroline-5-carboxylate dehydrogenase (*BpP5CDH*) and *6*-phosphogluconate dehydrogenase (*Bp6PGDH*), *BpTIFY5A*, Zeaxanthin epoxidase gene1 (*BpZEP1*), and *BpCAT*. All genes were up-regulated in the OE line except for *TIFY5A* and *P5CDH*, which were up-regulated in the Ri line (Figure 6). Genes related to ROS scavenging *BpSOD2*, *BpAPX1*, *BpAPX2*, *BpCAT*, and *Bp6PGDH* were significantly up-regulated after salt treatment, with the expression level of *BpSOD2* after salt stress reaching as much as 58-fold of that under control conditions in the OE line (Figures 6A–E). *Bp6PGDH* expression in the OE lines was up-regulated by 41.4-fold under salt treatment (Figure 6C), while the expression of *BpAPX1*, *BpAPX2*, and *BpCAT* was up-regulated by 4.32-, 13.02-, and 7.05-fold, receptively, under salt treatment in the OE line (Figures 6A,B,D). Similarly, *BpP5CS*, which participated in proline biosynthesis was up-regulated by 34.18-fold under salt conditions in the OE line (Figure 6H), whereas expression of *BpP5CDH*, a participant in proline degradation, was up-regulated by 3.7-fold in the Ri line and remarkably down-regulated in OE the line after salt treatment (Figure 6G). Meanwhile, expression of *BpTIFY5A*, a suppressor of JA responses, was also up-regulated in the Ri line, reaching as much as 9.95-fold that under control conditions after salt treatment (Figure 6I). Notably, *BpZEP1* is an important participant in ABA biosynthesis and was significantly down-regulated in the Ri line after salt stress, however, expression of *BpZEP1* showed no difference under control or salt-treated conditions in the OE line and the WT (Figure 6F). These results showed that *BpSZA1* has a positive function in response to salt stress. All primers used for RT-PCR analysis are detailed in Supplementary Table 5.

### Identification of Target Genes Directly Regulated by *BpSZA1*

**FIGURE 7:**
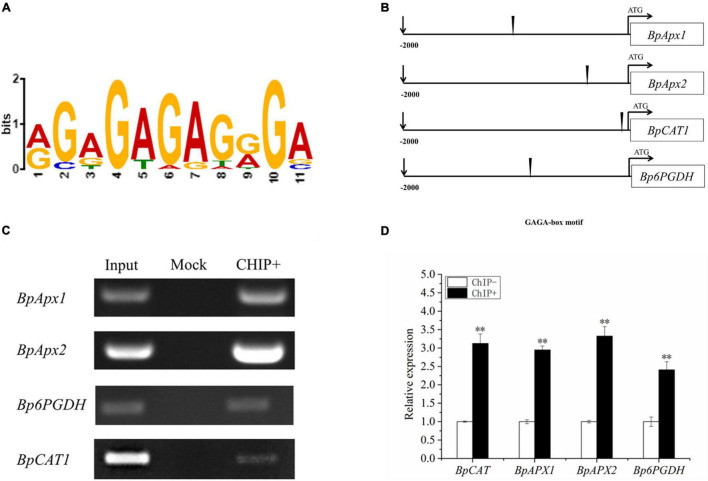
**(A)** Multiple Expectation maximizations for Motif Elicitation (MEME) analysis of the conserved sequence GAGA-box present in the promoters of genes regulated by *BpSZA1*. **(B)** The schematic diagram shows the positions of the GAGA-box in the promoters. **(C)** Enrichment of the promoters of genes containing GAGA-box. **(D)** ChIP-qPCR analysis shows enrichment of the promoter sequences of genes after ChIP. ChIP+: the sonicated chromatin was immunoprecipitated with GFP antibody; mock: the sonicated chromatin was immunoprecipitated with IgG2a; input: the sonicated chromatin was used as a positive control. Three biological replicates were performed. Asterisks denote significant differences: ^**^P ≤ 0.01. Error bars represent standard error for three replicates.

To identify the motif that binds *BpSZA1* for activating gene transcription following exposure to abiotic stress, we applied the MEME motif discovery tool (see text footnote 5). The promoters of 20 genes based on the qRT-PCR and RNA-seq data, that were up-regulated by *BpSZA1* were used for further study. MEME results revealed that there was an 11-base conserved sequence present in most of the promoters studied (Figure 7B). The fourth to seventh bases of the conserved sequences appeared with the highest frequency and might be the core sequences of this motif; therefore, they were used for further experiments (Figure 7A). The 11-base conserved sequences were named the GAGA-motif (AGAGAGAGGGA, occupy the highest proportio). ChIP-PCR and ChIP-qPCR demonstrated that *BpSZA1* protein bound to the GAGA-motif in the promoters of *BpAPX1*, *BpAPX2*, *BpCAT*, and *Bp6PGDH* (Figures 7C,D), indicating that the GAGA-motif combines with BpSZA1 to regulate the expression of *BpAPX1*, *BpAPX2*, *BpCAT*, and *Bp6PGDH* under salt stress. In addition, ChIP results indicated that genes associated with ROS scavenging ability, such as *BpAPX1*, *BpAPX2*, *BpCAT*, and *Bp6PGDH*, were predominantly regulated directly by *BpSZA1*.

## Discussion

### *BpSZA1* Belongs to C2H2 Zinc Finger Proteins and Confers Salt Tolerance

Multiple sequence alignment revealed that *BpSZA1* clustered with *AtAZF2*, *AtAZF3*, *AtZAT6*, *AtZAT10*, and *AtZAT13* from *Arabidopsis* (Figure 1B). Many studies have shown that important information on these genes plays an important role in the response to abiotic stress. According to reports in 2011, AZF1-OE and AZF2-OE lines in *Arabidopsis* show significant growth inhibition and sensitivity to abiotic stress (Kodaira et al., 2011). *AtZAT6* not only participates in root development but also increases tolerance to cadmium in transgenic *Arabidopsis* (Chen et al., 2016). Among poplars, ZAT10 OE lines display increased cold and salt tolerance compared with WT plants (He et al., 2019, 2020). In the current study, we found that *BpSZA1* is induced by salt. In addition, *BpSZA1* plays a positive role in the response of birch plants to salt stress. Many physiological parameters, including POD activity (Figure 5F), SOD activity (Figure 5G), and proline content (Figure 4C), are modulated through the expression of *BpSZA1*. Overall, these results indicate that BpSZA1 belongs to C2H2 ZFPs and positively modulates salt stress in birch.

### *BpSZA1* Binds to GAGA-Motif to Regulate Gene Expression

Transcription factors usually regulate downstream gene expression by combining with *cis*-acting elements, so it is important to identify which *cis*-element combines with each TF when studying the function of TFs. Utilizing MEME, we found that *BpAPX1*, *BpAPX2*, *BpCAT*, and *Bp6PGDH* all contain the GAGA-rich motif sequence (Figure 7A). Further, ChIP-PCR and ChIP-qPCR analysis showed that *BpSZA1* can directly regulate the expression of these genes by binding to GAGA-rich motifs in their promoters (Figures 7C,D). This result is consistent with those of previous studies. In *Drosophila*, the *Tr1* gene encodes a protein with a C2H2 domain that can bind to a GAGA element to regulate the expression of downstream genes (Pedone et al., 1996). A similar mechanism may exist in plants. RAMOSA1, with a single C2H2 ZFP domain, can bind to the GAGA element to regulate the inflorescence structure of maize (Eveland et al., 2014). Furthermore, it was recently predicted that *MaC2H2-4* and *MaC2H2-5* in bananas can regulate the ripening of banana fruits by combining with different TF binding sites, including two GA-rich motifs (Kuang et al., 2021). In summary, these results show that *BpSZA1* may regulate downstream gene expression by binding to the GAGA-repeat sequence.

### *BpSZA1* Enhanced Salt Tolerance by Directly Regulating Genes Related to Reactive Oxygen Species Scavenging

Abiotic stress including high salt and drought, causes apoplast oxidative stress in plants, accelerating the production of ROS including H_2_O_2_, ⋅O_2_^–^, ^1^O_2_, and ⋅OH^–^, which can damage and kill plants (Mittler et al., 2004; Suzuki et al., 2012; Baxter et al., 2014). However, the ROS generated can be scavenged by numerous enzymes. Major ROS-scavenging enzymes include SOD, APX, CAT, glutathione peroxidase (GPX), and peroxiredoxin (PrxR) in plants (Nakano and Asada, 1981; Rizhsky et al., 2004; Yang and Guo, 2018). In previous studies, *ZAT10* OE lines displayed enhanced salt tolerance by directly regulating the expression of *APX2* (Mittler et al., 2006; He et al., 2019). This result is consistent with our findings. *BpSZA1* bound to *BpAPX1* and *BpAPX2* promoter regions containing the GAGA-motif, and expression of *BpAPX1*, *BpAPX2* in the OE line was significantly increased after salt stress compared with that in WT and Ri line (Figures 6A,B), which may improve the salt tolerance of plants. Another important finding was that *BpSZA1* could directly bind to the GAGA-motif of the *BpCAT* promoter region (Figures 7C,D). At the same time, expression levels of *BpCAT* in OE lines and the WT were highly up-regulated after salt treatment compared with those under control conditions, but the expression level in the Ri lines was lower (Figure 6D). This is also in accordance with earlier observations, which showed that *NtERF172*, a TF of the AP2 family, also binds to the *NtCAT* promoter region to activate *NtCAT* transcription, and overexpression lines of *NtERF172* displayed greater ROS scavenging and better drought tolerance (Zhao et al., 2020). Furthermore, we found no significant difference in the expression of *BpSOD2* among WT, OE, and Ri lines. However, after salt treatment, expression levels were 1.5-fold higher than that in the WT and 58-fold than in the Ri line (Figure 6E). This indicates that *BpSZA1* could enhance salt tolerance by regulating the expression of *BpSOD2*.

Besides ROS-scavenging enzymes, the antioxidants ascorbic acid and glutathione can scavenge ROS produced in the stroma (Yang and Guo, 2018). The reduced coenzyme NADPH is an electron donor in the ascorbate-glutathione biosynthesis phase and is important for maintaining ascorbic acid and glutathione in a reduced state (Corpas et al., 1998). *6PGDH*, *G6DPH*, malic enzyme, and isocitrate dehydrogenases are the main cellular sources of NADPH (Mittler et al., 2004; Marino et al., 2012; Suzuki et al., 2012; Baxter et al., 2014). We found that *BpSZA1* could bind to the *Bp6PGDH* promoter region containing a GAGA-motif. The expression level of *Bp6PGDH* in OE lines after salt stress was 40-fold higher than that without salt stress. However, there was no significant difference in *Bp6PGDH* expression in WT or Ri lines between salt treatment and control conditions (Figure 6C). This finding has also been reported in previous research. Salinity-tolerant lines of barley have a higher expression level of *6PGDH* (Witzel et al., 2010). Overexpression of *BpSZA1* conferring tolerance to salt might increase the expression of *Bp6PGDH*. *BpSZA1* might therefore enhance salt tolerance by regulating genes related to ROS scavenging, including *BpAPX1*, *BpAPX2*, *BpCAT*, *BpSOD2*, and *Bp6PGDH*.

### *BpSZA1* Improves Salt Tolerance by Participating in Abscisic Acid Synthesis, Proline Synthesis, and Activating the Abscisic Acid/Jasmonic Acid Pathways

The gene ZEP1 is a key gene for plant biosynthesis of ABA (Zhu, 2002). In 1998, the ZEP gene was reported to be involved in the biosynthesis of ABA precursors (Audran et al., 1998). Meanwhile, in a recent study, a *ZEP* gene *MsZEP* from alfalfa (*Medicago sativa*), overexpression of *MsZEP* enhances drought and salt tolerance by increasing ABA biosynthesis (Zhang et al., 2016). This is consistent with our study. After salt stress, the expression level of ZEP1 in the Ri lines was lower than that in WT plants; however, the expression level of ZEP1 in the OE lines and WT plants did not vary significantly before and after the stress. This shows that the absence of *BpSZA1* might inhibit the activity of the *BpZEP1* enzyme and affect the ABA-dependent pathway response of plants to salt stress (Figure 6F).

Proline is a component of plant proteins and has a critical role in regulating cell redox potential (Kavi Kishor et al., 1995; Zhu, 2002). In this study, expression levels of some genes related to proline biosyntheses, such as *BpP5CS* (Figure 6H) and *BpP5CDH* (Figure 6G), were significantly increased in OE lines after salt stress. At the same time, proline content was also increased after salt stress in OE lines, balancing the osmotic pressure inside and outside the cell and protecting the cell from damage. This indicates that *BpSZA1* positively regulates proline biosynthesis to improve the salt tolerance of plants.

It is well documented that because ZFPs contain an EAR motif, they may act as transcriptional repressors in regulating the tolerance of plants to abiotic stress (Ciftci-Yilmaz et al., 2007; Xie et al., 2019; Wang et al., 2020). We found that only the expression level of *TIFY5A/JAZ8* in Ri lines increased instantaneously after salt stress, indicating that *BpSZA1* might inhibit the expression of *TIFY5A/JAZ8*. *TIFY5A* belongs to the JAZ subfamily of the TIFY TF family and is also known as JAZ8 (Shyu et al., 2012). It has previously been observed that ZAT10 can regulate the expression of *TIFY10a/JAZ1* in the JA signaling cascade (Pauwels et al., 2008). When not responding to the JA signal, JAZ proteins combine with and inhibit downstream TFs expression to suppress JA responses. After being induced by JA signals, JAZ proteins are ubiquitinated and degraded through the 26S proteasome, thereby activating downstream TF transcription and JA responses (Fernandez-Calvo et al., 2011; Ruan et al., 2019; Yang et al., 2019). JA participates in plant salt responses. Extensive research has shown that the exogenous application of JA enhances salt tolerance by maintaining ROS or ion homeostasis (Qiu et al., 2014; Jiang et al., 2016; Farhangi-Abriz and Ghassemi-Golezani, 2018; Tavallali and Karimi, 2019). Overall, this indicates that inhibiting *BpSZA1* expression may increase the expression level of *BpTIFY5A* and further suppress JA responses, thereby destroying ROS homeostasis and ion homeostasis and resulting in Ri lines showing more sensitivity to salt treatment (Figure 6I). Consequently, BpSZA1 may enhance the salt tolerance of plants by participating in JA responses. However, how BpSZA1 responds to salt stress through involvement in the JA signaling pathway requires further study.

## Conclusion

**FIGURE 8:**
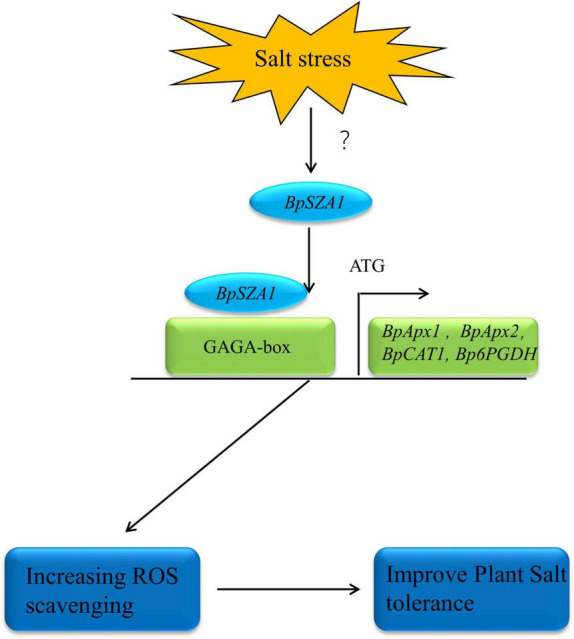
The model of BpSZA1 regulation network in response to Salt stress. Salt stress signals activate the expression of upstream genes of BpSZA1, which in turn activates *BpSZA1*. *BpSZA1* binds to the GAGA-box in the promoter region, thereby activating the expression of downstream genes such as *BpAPX1*, *BpAPX2*, *BpCAT*, and *Bp6PGDH* to increase ROS scavenging. The result is tolerance to salt stress is increased in the plants.

In summary, we identified a novel C2H2 ZFP, *BpSZA1*, which plays a positive role in the plant salt stress response. We analyzed the function of *BpSZA1* by generating OE and Ri in transgenic birch plants. We further showed that BpSZA1 can bind directly to the GAGA-motif in the promoter regions of various genes related to ROS scavenging to regulate their expression and maintain ROS homeostasis (Figure 8). Thus, our findings provide new insights into the function of C2H2 ZFPs in abiotic stress and further efforts to cultivate salt-tolerant birch varieties.

## Data Availability Statement

The original contributions presented in the study are publicly available. This data can be found here: NCBI, SRA, PRJNA808444.

## Author Contributions

LL designed the research. XZ, QG, and LQ conducted the experiments and data analysis. XZ wrote the manuscript and performed the transcriptome data analysis. All authors read and approved the manuscript.

## Conflict of Interest

The authors declare that the research was conducted in the absence of any commercial or financial relationships that could be construed as a potential conflict of interest.

## Publisher’s Note

All claims expressed in this article are solely those of the authors and do not necessarily represent those of their affiliated organizations, or those of the publisher, the editors and the reviewers. Any product that may be evaluated in this article, or claim that may be made by its manufacturer, is not guaranteed or endorsed by the publisher.

## Footnotes

1
http://web.expasy.org/cgi-bin/protparam/

2
https://www.arabidopsis.org

3
https://phytozome.jgi.doe.gov/pz/portal.html

4
https://genomevolution.org/coge/GenomeInfo.pl?gid=35079

5
http://meme-suite.org/

6
http://ccb.jhu.edu/software/hisat2/index.shtml

7
http://tophat.cbcb.umd.edu/

8
https://ccb.jhu.edu/software/stringtie/index.shtml

9
https://www.biostat.wisc.edu/∼kendzior/EBSEQ/
