# Supplementary material for: A Cys2His2 Zinc Finger Transcription Factor BpSZA1 Positively Modulates Salt Stress in Betula platyphylla
Source: Front Plant Sci. 2022 May 25;13:823547. doi: 10.3389/fpls.2022.823547 (PMC9174930; doi:10.3389/fpls.2022.823547)
Supplement: Supplementary file 1 [file Data_Sheet_1.zip › Table 1.docx]

Figure S1


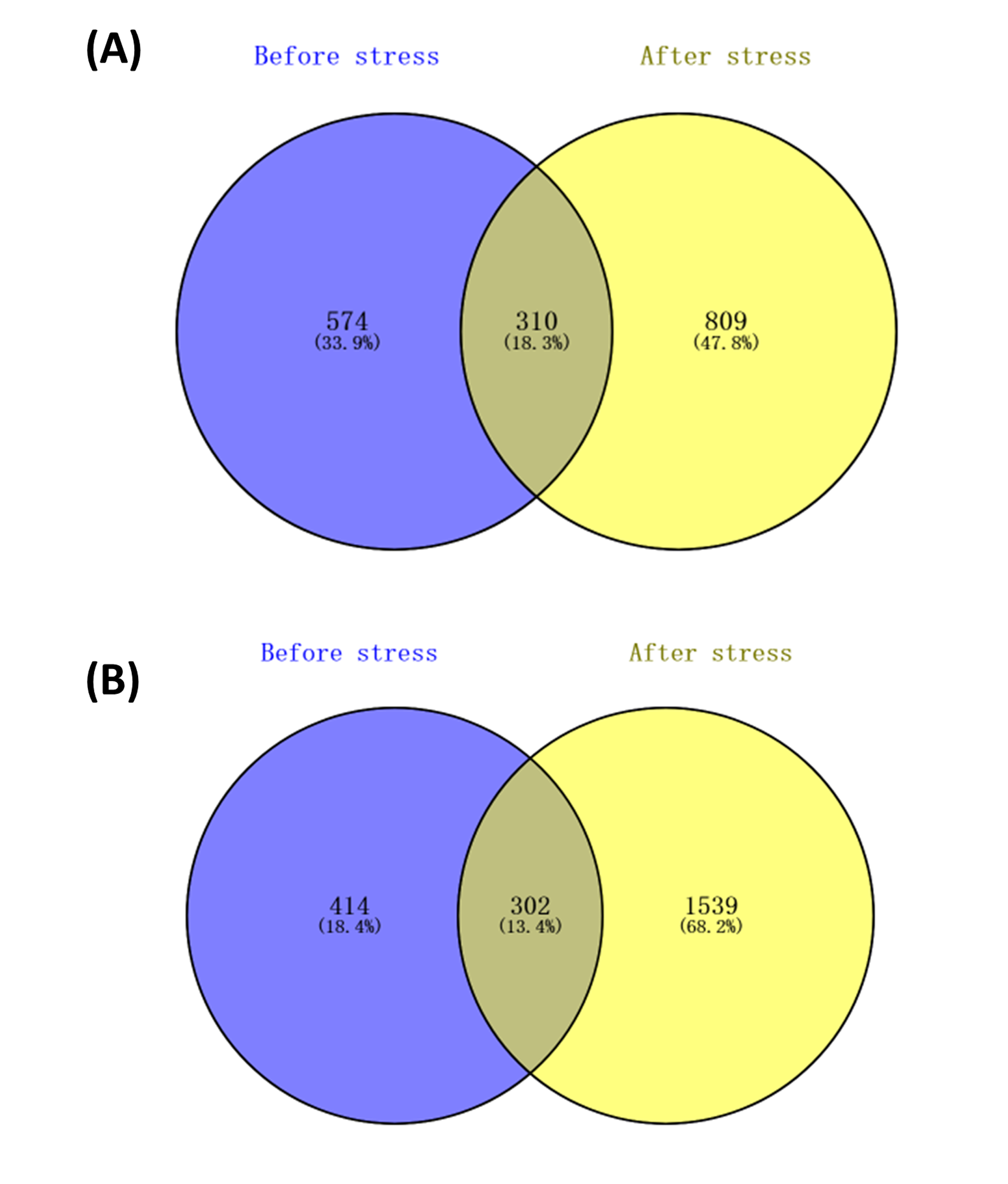


**Figure S1**. Differentially expressed genes of up-regulated or down-regulated genes affected by *BpSZA1* under salt stress. (**A**) Upregulated genes affected by *BpSZA1* before and after salt stress. (**B**) downregulated genes affected by *BpSZA1* before and after salt stress.

Figure S2


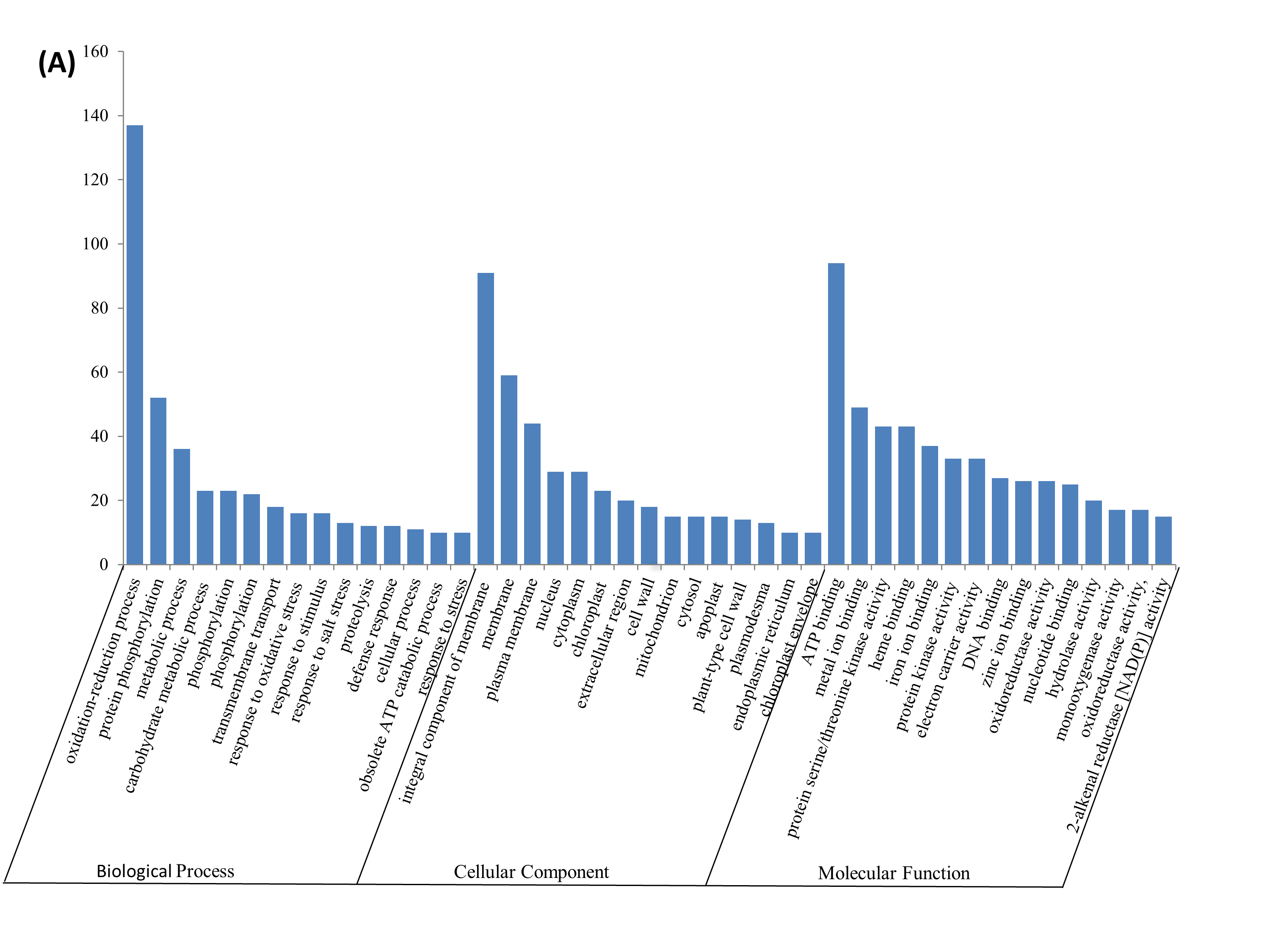

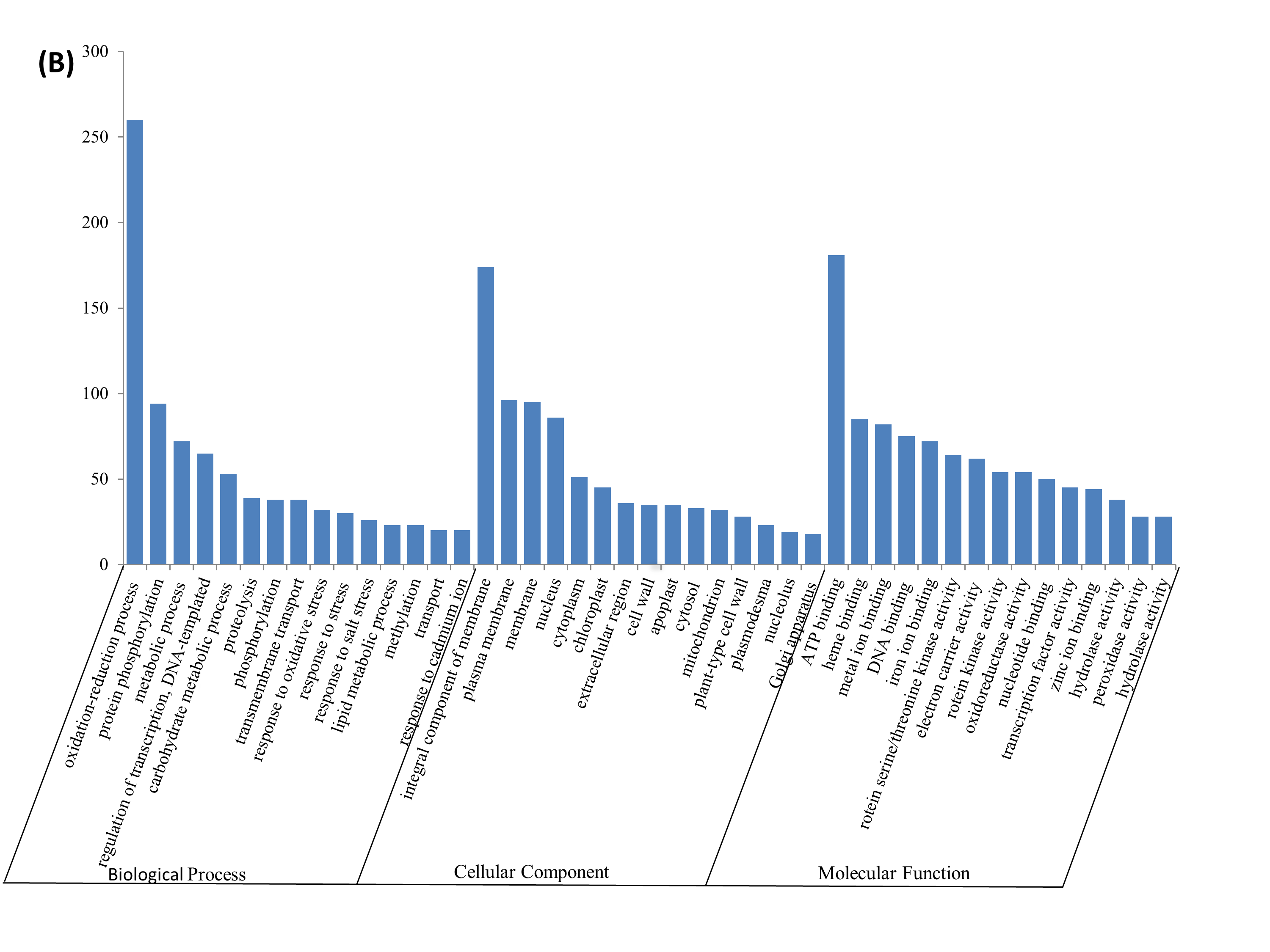


**Figure S1**. Differential gene GO enrichment of transgenic plants compared with wild-type plants (**A**) Genes before salt stress and (B) Genes after salt stress and
