# Supplementary material for: A Cys2His2 Zinc Finger Transcription Factor BpSZA1 Positively Modulates Salt Stress in Betula platyphylla
Source: Front Plant Sci. 2022 May 25;13:823547. doi: 10.3389/fpls.2022.823547 (PMC9174930; doi:10.3389/fpls.2022.823547)
Supplement: Supplementary file 1 [file Data_Sheet_1.zip › Table 2.docx]

**Table S1 Primer sequences used in the analysis of *BpSZA1* target genes using real-time**

| Primer | Sequence (5’-3’) |
| --- | --- |
| BpSZA1-RT-F | AAGCGTTGTCACTACGAAGG |
| BpSZA1-Rt-R | TAGGCGAGAATTCCGGCAAG |
| BpTubulin-F | TCAACCGCCTTGTCTCTCAGG |
| BpTubulin-R | TGGCTCGAATGCACTGTTGG |

**Table S2 Primer sequences used in the construction of *BpSZA1* overexpression and silencing lines.**

| Primer | Sequence (5’-3’) |
| --- | --- |
| BpSZA1-F | CACCATGGCTCTTGAAGCTCTCAACTC |
| BpSZA1-R | ATGTTGAGAGATTTCGATCTTC |
| BpSZA1-RNAi-F | CACCAGCAGCAGCGTCGTAACAACCT |
| BpSZA1-RNAi-R | ATGTTGAGAGATTTCGATCTTC |
| BpSZA1-Ri-F-Reverse | AGGTTGTTACGACGCTGCTGCT |
| LR-35S primer-F | CCTCGGATTCCATTGCCCAGCTA |
| LR-GFP primer-R | GTCGATGCCCTTCAGCTCGAT |
| pB7G-35S | GACGCACAATCCCACTATCC |
| pB7G-35S-Terminate | GCTCAACACATGAGCGAAAC |

**Table S3 Primer sequences used in the construction of 35S:BpSZA1-GFP**

| Primer | Sequence (5’-3’) |
| --- | --- |
| BpSZA1-GFP-F | TGCTCTAGAATGGCTCTTGAAGCTCTCAACTC |
| BpSZA1-GFP-R | CCCCCGGGTAATGTTGAGAGATTTCGATCTTC |
| pBI121-F | CCATCGTTGAAGATGCCTCTGC |
| pBI121-R | CGCAAGACCGGCAACAGGAT |

**Table S4 Primer sequences used to amplify the whole or truncated CDS of *BpSZA1* in the transactivation assay.**

| Primer | Sequence (5’-3’) |
| --- | --- |
| BpSZA1-BD-F | CATGCCATGGAGATGGCTCTTGAAGCTCTCAA |
| BpSZA1-BD-R | CGGGATCCATGTTGAGAGATTTCGATCTTCAATG |
| BpSZA1-388-F | CATGGAGGCCGAATTCATGAGGAAGCCGGAGGCCT |
| BpSZA1-387-R | GCAGGTCGACGGATCCCATATACATCATGGGCATC |
| BpSZA1-526-F | CATGGAGGCCGAATTCGATTCATCATCATCATCAT |
| BpSZA1-525-R | GCAGGTCGACGGATCCCTGAACTTGGAAATCAAG |
| BpSZA1-633-R | CATGGAGGCCGAATTCATGAGGAAGCCGGAGGCCT |
| BpSZA1-634-F | GCAGGTCGACGGATCCCATGTTAAGGGCGCCATGC |
| BpSZA1-690-R | CATGGAGGCCGAATTCTCAACCGGATATGATCTT |
| BpSZA1-691-F | GCAGGTCGACGGATCCCTGAACTTGGAAATCAAG |

**Table S5 Primer sequences used in the analysis of *BpSZA1* target genes using real-time RT-PCR.**

| Primer | Sequence (5’-3’) |
| --- | --- |
| APX1F | TCAAATCACAGCCCAAAG |
| APX1R | CTCCTCCGAGTGCTTCAT |
| APX2F | AAGAAGTATCCAACCGTGAGC |
| APX2R | ACTGGTAGAAATCGGCGTAG |
| SOD2-F | GACTACTGGGAATGCTGGTG |
| SOD2-R | CAATCGAGCCTAAACGGACT |
| 6PGDH-F | TCTGAACTTCGGTTTCTTGT |
| 6PGDH-R | GTATGCCAAATCAGTGCTAA |
| P5C-F | TTCAGCCTCCAACACCTCAT |
| P5C-R | ACCAGCCTAACCAACCCATC |
| P5CDH-F | TCTATACCCTTTGCTACCGT |
| P5CDH-R | ATGCCTGTTTCATCTACCTC |
| ZEP-F | ATCACGGATATTGAGGAC |
| ZEP-R | TCATACTGTCTGGACGATT |
| CAT-F | GAGGTGGAGATGATACGT |
| CAT-R | GGGCATAAAGCCAGATAA |
| TIFY5A-F | AGGAAGAGGAAACCCGAAAC |
| TIFY5A-R | TAGGGCTACATGCCGGAGAT |
| BpTubulin-F | ATGATGATCACCGTGCGTG |
| BpTubulin-R | CGTTGAGCATGAAGATCCAGAAG |

**Table S6 Primer sequences used in ChIP-PCR and ChIP-qPCR analysis**

| Primer | Sequence (5’-3’) | Length of amplification (bp) |
| --- | --- | --- |
| APX1-ChIP-F | GCTGCGGCGTTGAGTGTTG | 290 |
| APX1-ChIP-R | ACGTCATTTGGCTTGGAAC |  |
| APX2-ChIP-F | GGTCGTTCAAACCAATCTC | 340 |
| APX2-ChIP-R | CCACGCTAATCTAAATCTACAA |  |
| 6PGDH-ChIP-F | AGTGGATCTAGGATTAAGGGATT | 376 |
| 6PGDH-ChIP-R | GTGGCAGAAAGGAGTAAGAAACT |  |
| CAT-ChIP-F | GCTTCGTCTCCATTTCCAC | 130 |
| CAT-ChIP-R | TGCAAATCCATGCATCAAT |  |
| α-tubulin-F | GCATTCTGATGCCATTTC | 191 |
| α-tubulin-R | CAAGAAGGCCGCCACCAG |  |

**Table S7 Specific motif sequences of *BpSZA1* and five C2H2-type transcription factor C1-2i subfamily members in Arabidopsis thaliana.**

| Primer | Sequence (5’-3’) |
| --- | --- |
| Motif 1 | 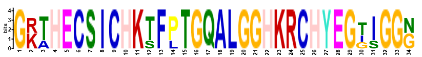 |
| Motif 2 | 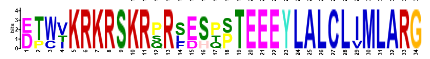 |
| Motif 3 | 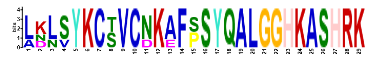 |
| Motif 4 | 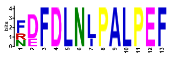 |
| Motif 5 | 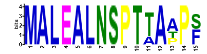 |
